# Supplementary material for: Intracellular Water Content in Lean Mass is Associated with Muscle Strength, Functional Capacity, and Frailty in Community-Dwelling Elderly Individuals. A Cross-Sectional Study
Source: Nutrients. 2019 Mar 19;11(3):661. doi: 10.3390/nu11030661 (PMC6471552; doi:10.3390/nu11030661)
Supplement: Supplementary file 1 [file nutrients-11-00661-s001.pdf]

Table S1. Relationship between Lean Mass (kg) and indicators of muscle strength and functional capacity (continuous variables).

	r_s	p	β*	p
Hand grip (kg)	0.751	**<0.001**	0.768	**<0.001**
Barthel score	0.225	**<0.001**	0.160	**<0.001**
Gait speed (m/sec)	0.260	**<0.001**	0.006	**<0.001**
TUG (sec)	-0.250	**<0.001**	-0.065	**0.001**
Outdoor walking (hr/day)	0.156	**0.008**	0.531	**0.022**

* ICW/LM ratio as the independent variable.ICW, intracellular water; LM, lean mass; TUG, timed up-and-go.r_s, Spearman correlation coefficient; β, linear regression coefficient; Bold indicate statistically significant results.

Table S2. Relationship between Lean Mass (kg) and indicators of functional capacity and frailty (categorical variables).

	Mean; SD (N) Lean Mass when condition present	Mean; SD (N) Lean Mass when condition not present	p	Effect size
Frailty	44.8; 9.0 (46)	48.9; 8.5 (278)	**0.003**	-0,48
Weight loss	45.4; 8.4 (16)	48.5; 8.6 (308)	**0.127**	-0,36
Exhaustion	44.4; 8.3 (64)	49.3; 8.5 (260)	**<0.001**	-0,58
Poor muscle strength	45.4; 8.3 (104)	49.7; 8.5 (220)	**<0.001**	-0,51
Poor gait speed	44.7; 8.5 (67)	49.3; 8.4 (257)	**<0.001**	-0,55
Poor physical activity	45.4; 8.4 (100)	49.6; 8.5 (224)	**<0.001**	-0,49
Outdoor life	48.7; 8.5 (288)	45.2; 9.2 (35)	**0.021**	0,38
Unable to stand on 1 foot for 5 sec	49.1; 8.3 (233)	46.4; 9.1 (91)	**0.012**	0,33
Poor physical activity	45.6; 9.1 (81)	49.2; 8.3 (243)	**0.001**	-0,43

ICW, intracellular water; LM, lean mass; SD, standard deviation; effect size, (mean of the group with the condition – mean of the group without the condition)/standard deviation of the group with the condition; Bold indicate statistically significant results.
